# Predictors of Significant Liver Fibrosis in People with Chronic Hepatitis C Who Inject Drugs in the Czech Republic

**DOI:** 10.3390/life13040932

**Published:** 2023-04-02

**Authors:** Sona Frankova, Nikola Uzlova, Dusan Merta, Veronika Pitova, Jan Sperl

**Affiliations:** 1 Department of Hepatogastroenterology, Institute for Clinical and Experimental Medicine, 14021 Prague, Czech Republic; 2 Department of Internal Medicine, University Hospital Kralovske Vinohrady, 10034 Prague, Czech Republic; 3 Third Faculty of Medicine, Charles University, 10034 Prague, Czech Republic; 4 Cardiothoracic Anaesthesiology and Intensive Care, Department of Anaesthesiology and Intensive Care Medicine, Institute for Clinical and Experimental Medicine, 14021 Prague, Czech Republic; 5 First Faculty of Medicine, Charles University, 12108 Prague, Czech Republic

**Keywords:** chronic hepatitis C, significant liver fibrosis, obesity, harmful drinking, age, people who inject drugs

## Abstract

Background and objectives: HCV infection often remains untreated in people who inject drugs (PWID), albeit they may present with advanced liver fibrosis at a young age. We aimed to assess the rate of patients with significant fibrosis in PWID starting anti-HCV therapy and identify the factors associated with severe fibrosis. Methods: The cohort of 200 patients was divided into two groups: F0–F2 (N = 154, 77%), patients with liver stiffness measurement (LSM) < 10.0 kPa, and F3–F4 (N = 46, 23%), with LSM ≥ 10.0 kPa, indicating significant liver fibrosis. Results: In group F3–F4, there were significantly more males, and the patients were older, with a higher BMI. The number of long-term abstaining patients was significantly higher in group F3–F4 compared with group F0–F2, as well as the proportion of patients reporting harmful drinking. Obesity (OR 4.77), long-term abstinence from illicit drugs (OR 4.06), harmful drinking (OR 2.83), and older age (OR 1.17) were significant predictors of advanced fibrosis in PWID starting anti-HCV therapy. Conclusions: A quarter of PWID presented with significant liver fibrosis at treatment initiation. Obesity, long-term drug abstinence, harmful drinking, and older age contributed to significant liver fibrosis.

## 1. Introduction

Globally, 58.6 million people are estimated to be infected with HCV [1]. Being the most prevalent blood-borne infection in people who inject drugs (PWID), chronic hepatitis C represents the most important yet easily preventable cause of liver morbidity and mortality in these individuals, with an estimated anti-HCV positivity rate in Europe of 53.2–64.7% [2,3,4,5]. The risk of contracting HCV infection increases with drug injection duration [6], but recent data show that many PWID get infected early in their drug injection career [7]. The treatment of hepatitis C in the patients’ groups with its high prevalence is essential not only to prevent the progression of liver disease in a particular patient but also to reduce the further spread of the infection in so-far healthy individuals, especially in the PWID community [8]. In high-income countries, PWID represent the core of the HCV epidemic, with a high potential to sustain the epidemic through high-risk behaviour with ongoing virus transmission [5]. 

In the interferon era, active PWID were excluded from nearly all clinical trials of HCV treatment. Moreover, in real clinical practice, the treatment uptake among injection drug users was limited owing to poor treatment tolerability and its low efficacy [9]. A high proportion of patients presented with a contraindication to therapy, such as a psychiatric disorder or other severe comorbidities. Remaining in a 48-week treatment was demanding, with regular blood testing and injection application of the antiviral drugs.

Although the advent of direct-acting antivirals has made HCV infection curable in most patients who have access to and initiate antiviral therapy, PWID often remain largely untreated due to many reasons—e.g., a presumption of low efficacy owing to an impaired adherence to therapy and an anticipated high risk of reinfection due to ongoing risk behaviour. Therefore, when referred to secondary and tertiary treatment centres, PWID can be a priori stigmatised, and antiviral treatment can be refused or postponed. The PWID referred to therapy are usually younger than those with no drug use history [10], which can lead to a false presumption of an absence of advanced liver disease, justifying treatment deferral. 

The Czech Republic is a country with a low prevalence of HCV infection (less than 1% of the population), with the peak prevalence in the age group between 30 and 45 years for both males and females [2]. Still, intravenous drug use represents the most common cause of HCV transmission nowadays, and PWID represent more than 80% of patients referred to antiviral therapy [11]. Furthermore, in the Czech Republic, PWID are considered difficult to treat. Still today, many physicians revere the moral model of addiction and consider drug addiction to be a moral failure which should be punished. Long-term documented abstinence from illicit drugs is often required in treatment centres in order to prevent HCV reinfection after a successful course of therapy. Many visits to medical centres to prove appropriate adherence prior to treatment initiation postpone it. Nowadays, the test-and-treat strategy is preferred; the treatment should be initiated without delay after HCV is diagnosed [11,12]. 

The risk factors contributing significantly to the progression of liver disease in hepatitis C have been elaborately described in the past: older age at the time of infection, harmful drinking, male sex, and infection with genotype 3 [13,14]. Despite the young age, left untreated, patients with ongoing drug use can present with significant liver fibrosis at the time of diagnosis of HCV infection, and early treatment initiation seems to be vital to avoid cirrhosis decompensation and HCC occurrence. 

The aim of our study was to assess the rate of patients with significant liver fibrosis in HCV-infected PWID referred to antiviral therapy and identify the factors predicting the presence of significant liver fibrosis and cirrhosis in this patient population.

## 2. Materials and Methods

From a total number of 643 patients with HCV infection starting antiviral therapy, two hundred (31.1%) consecutive adult patients with a history of past or current intravenous drug use who initiated anti-HCV therapy at the outpatient clinic of the Department of Hepatogastroenterology at the Institute for Clinical and Experimental Medicine, Prague, Czech Republic, were enrolled in the study between January 2017 and December 2019. The patients were referred to our tertiary centre by infectious disease specialists, gastroenterologists, general practitioners, psychiatrists, and addiction specialists. Forty-seven (23.5%) patients came without a referral letter, addressed by public-awareness campaigns. Before treatment initiation, all patients underwent a laboratory assessment of liver function tests, HCV RNA levels, and HCV genotype. Abdominal ultrasound was performed as an HCC screening examination. In all patients, liver stiffness (LSM) was measured by Vibration-Controlled Transient Elastography (VCTE, FibroScan^®^, Echosens, France) according to the manufacturer’s recommendation to evaluate the stage of liver fibrosis together with Controlled Attenuation Parameter (CAP, FibroScan^®^, Echosens, France) to assess the grade of steatosis. According to LSM results, the cohort was divided into two groups:

- Group F0–F2 (N = 154), including patients with LSM less than 10.0 kPa (no fibrosis < 5 kPa, mild fibrosis 5–7 kPa, moderate fibrosis > 7 kPa);
- Group F3–F4 (N = 46), with LSM 10.0 kPa and more, consisting of patients with severe fibrosis (F3, 10.1–12.5 kPa) and cirrhosis (F4, >12.5 kPa), considered to be patients with significant liver fibrosis in the following analysis [15].

The demographic data were recorded at the patient’s first visit and updated appropriately. 

Alcohol consumption was regarded as harmful drinking when the patient reported a daily alcohol intake of more than 10 g of pure alcohol for women and 20 g for men [16]. The length of abstinence from illicit drug use was self-reported: ongoing or recent drug use, abstinence for more than one year, and abstinence for more than five years. Neither blood nor urine drug screen tests were performed.

The patients’ data were extracted retrospectively from the electronic hospital patients’ database; the recording of medical history was based on a uniform template and conducted by two department physicians (J.S., S.F.). All the data were recorded in the outpatient visit files. 

After successful antiviral therapy, the patients from group F0–F2 were dismissed from further follow-up after one year, owing to negligible further risk of HCV complications. The patients in group F3–F4 were enrolled in the HCC surveillance follow-up, according to current guidelines [12], which recommend an ultrasound examination biannually in patients with pre-treatment LSM of more than 10 kPa. Their follow-up adherence and occurrence of complications were assessed until 10 December 2022.

### 2.1. Antiviral Treatment

Antiviral treatment was started without delay when HCV RNA and HCV genotype were available, in most cases, at the first visit to the centre. All patients were treated with direct-acting antivirals. All administered regimens were all oral, and the duration of therapy was from 8 to 12 weeks. The treatment regimen was chosen according to the availability of the drugs on the market at the time of treatment initiation, HCV genotype, stage of liver disease, and renal function. Sustained virological response (SVR12), i.e., HCV cure, was assessed twelve weeks after the end of therapy as negative values of plasma HCV RNA. The antiviral regimens used are listed in Table 1.

### 2.2. Compliance with Ethical Standards

The study was approved by the Ethics Committee of Thomayer Hospital and the Institute for Clinical and Experimental Medicine, Prague, Czech Republic, and conducted in compliance with the Helsinki Declaration. The patients’ informed consent was not required because of the retrospective character of the study and the anonymous character of patients’ data.

### 2.3. Statistical Analysis

All statistical analyses were performed using the GraphPad Prism version 9.4.0 for macOS, GraphPad Software, San Diego, CA, USA. Clinical characteristics were analysed in a descriptive way and reported as medians and ranges. 

The Chi-square test or Fisher’s exact test were used for frequency analysis, according to the sample size: The Chi-square test was used to calculate HCV genotype frequencies, illicit drug use, history of imprisonment, smoking, reference to therapy, and education. Fisher’s exact test was used for the calculation of frequencies of sex, obesity, diabetes, HBV infection, opioid substitution therapy, history of harmful drinking, alcohol addiction treatment therapy, tattoo, history of psychiatric comorbidity. 

For continuous data, Mann–Whitney test was used, owing to the non-parametric distribution of the data, for age, BMI, ALT activity, GGT activity, and HCV RNA levels.

Factors predicting significant liver fibrosis were examined using multivariate logistic regression analysis. 

The *p* value < 0.05 was considered statistically significant throughout the study.

## 3. Results

### 3.1. Patient Demographic Characteristics

The detailed characteristics of the patients of the two study groups are shown in Table 2. The median age of the whole cohort was 38 years (range 25–62 years). In group F3–F4, there were significantly more males (80.4% vs. 63.0%, *p* = 0.032), the patients were significantly older (45 vs. 35 years, *p* < 0.0001), and they had a higher BMI (30 vs. 25, *p* < 0.0001) in comparison with group F0–F2. The BMI did not differ significantly between patients who reported ongoing or recent drug use and those who abstained for more than one year, 24.1 vs. 25.7, respectively (*p* = 0.26). 

In group F3–F4, the patients presented, in comparison with group F0–F2, with a higher activity of liver enzymes: ALT 136 vs. 82 IU/L (range 15–431 and 17–807 IU/L, respectively) and GGT 166 vs. 55 IU/L (range 33–1138 and 10–1220 IU/L, respectively) and had a significantly higher baseline HCV viral load, 1,305,000 vs. 512,000 IU/mL (range 5020–13,000,000 and 35–20,100,000, respectively). They also had significantly different HCV genotype distribution (HCV genotype 1b being the most prevalent in group F3–F4, 54.3%, *p* = 0.005). Together with LSM, CAP values were assessed. They were significantly higher in group F3–F4 than in group F0–F2 (276.5 vs. 242.5 dB/m, *p* = 0.0013) and corresponded to moderate and mild steatosis, respectively. 

### 3.2. Illicit Drug Use and Harmful Drinking

In group F3–F4, the overall number of patients who were long-term abstaining from intravenous drug use was significantly higher than in group F0–F2 (71.7% vs. 44.1%, *p* = 0.0017). The proportion of individuals with recent and ongoing drug use was significantly higher in group F0–F2 42/154 (27.3%) vs. 3/46 (6.6%) in group F3–F4, *p* = 0.0023. By contrast, the number of patients with reported abstinence for more than five years was significantly higher in group F3–F4 than in group F0–F2 (33/46, 71.7% vs. 68/154, 44.1%, respectively, *p* = 0.0013).

In group F3–F4, the proportion of patients reporting harmful drinking significantly differed from group F0–F2 (39.1% vs. 16.9%, *p* = 0.0023); they also reported a more frequent history of hospital alcohol addiction therapy (*p* = 0.044).

### 3.3. Anti-HCV Treatment Efficacy

Treatment efficacy was assessed as an intention to treat analysis; all patients who started treatment were included in the statistical analysis of treatment efficacy, as shown in Table 1. SVR12 was achieved in 92.0% of patients in group F0–F2 and 91.4% of patients in group F3–F4. The treatment efficacy did not differ and was excellent in both treatment groups. Thirteen patients (8.4%) in group F0–F2 and two (4.3%) in group F3–F4 were lost to follow-up; one patient relapsed (0.6%) in group F0–F2, and so did two patients (4.3%) in group F3–F4. One known case of HCV reinfection occurred in group F0–F2 during the first year after the end of therapy. All relapsed or reinfected patients thereafter underwent successful HCV retreatment.

### 3.4. Baseline Factors Predicting Significant Liver Fibrosis in PWID

Analysis of potential predictors of significant liver fibrosis was performed based on the data from univariate analysis (Table 2), and the following factors were further analysed: infection with HCV genotype 1b, sex, obesity (BMI > 30), the duration of drug abstinence of more than one year, harmful drinking, and age. 

In multivariate logistic regression analysis, neither the male sex (OR 1.28, 95% CI 0.50–3.46, *p* = 0.5) nor HCV genotype 1b (OR 0.91, 95% CI 0.38–2.13, *p* = 0.75) contributed significantly to significant liver fibrosis. 

**Figure 1:**
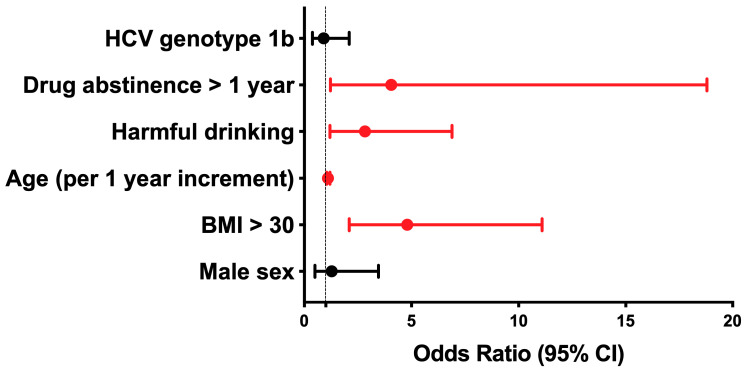
Predictors of significant fibrosis in PWID. Significant predictors are depicted in red. CI, Confidence Interval.

On the other hand, obesity (OR 4.77, 95% CI 2.1–11.1, *p* < 0.001), abstinence from illicit drugs for more than one year (OR 4.06, 95% CI 1.22–18.79, *p* < 0.03), harmful drinking (OR 2.83, 95% CI 1.17–6.89, *p* < 0.03), and older age (OR 1.17, 95% CI 1.05–1.17, *p* < 0.001) turned out to be significant predictors of an advanced stage of liver fibrosis at the time of treatment initiation (Figure 1). 

### 3.5. Follow-Up and HCC Surveillance in Group F3–4

The patients in group F0–F2 were dismissed from further follow-up one year after the end of therapy. Thirteen (8.4%) patients were lost to follow-up within that time period. All the patients were advised on harm reduction measures, including participation in needle syringe exchange programmes, and informed about other risks of transmission representing the potential sources of reinfection, such as unprotected sex, amateur tattoo, or imprisonment. The patients were also offered to get retested if they perceived their behaviour as risky.

According to current guidelines [12,17], the patients in group F3–F4 were enrolled in the HCC surveillance programme and underwent an ultrasound examination biannually. Eighteen cured patients were sent back to the referring centres for further follow-up. Twenty-eight patients continued their HCC surveillance at our centre, with a median follow-up period of 50.4 months. Nineteen patients (67.9%) remain in HCC surveillance. Nine (32.1%) patients were lost to follow-up at a median of 24 months. One patient with ongoing alcohol consumption died from acute alcoholic hepatitis, and one patient presented with HCC and was indicated for liver transplantation. One patient presented with decompensation of liver cirrhosis owing to a short period of alcohol consumption relapse; he recompensed after resuming alcohol abstinence. 

## 4. Discussion

In our study conducted on past or current PWID with hepatitis C initiating antiviral treatment, we found that 46 (23%) patients had fibrosis F3 or F4. Those patients were older, and their median age was 45 years. They more often reported harmful drinking, and more of them were obese. They had also abstained from illicit drug use for a longer period of time in comparison with patients with fibrosis F0–F2. Despite the young age of PWID who start antiviral treatment [11,18,19,20], the proportion of those who present with advanced liver disease is not negligible; Rosenthal et al. reported that 33% of patients with cirrhosis were starting anti-HCV therapy concurrently with opioid substitution therapy [18]. In the Simplify study [19], the rate of patients with significant liver fibrosis was 27%, assessed with vibration-controlled transient elastography in most of the patients. Kåberg et al. assessed the stage of liver fibrosis in a large cohort of PWID with chronic hepatitis C; out of 964 individuals, 15% presented with significant fibrosis (>9.5 kPa), and the authors pointed out the need for early referral to antiviral therapy [21]. 

Once HCV infection is diagnosed, antiviral treatment should not be deferred, despite the young age of these individuals. The factors associated with the progression of liver disease in hepatitis C have been described previously: older age at the time of diagnosis and the duration of the illness are associated with a higher degree of liver fibrosis [22]. An accelerated course is also associated with alcohol consumption, HIV and HBV co-infection, diabetes, obesity, and steatosis [23,24]. 

As many PWID get infected with HCV early in their drug injection career [7], the illicit drug abstinence period may serve as a “surrogate marker” of the duration of the infection. The longer the abstinence period, the longer the HCV infection duration contributing to fibrosis progression. Nowadays, most PWID who are in contact with harm reduction services are offered regular HCV screening. Contrarily, people who abandoned illicit drug use a long time ago may never have been tested and may present with advanced liver disease. Therefore, screening based on the history of risk factors for HCV transmission is of paramount importance. 

The duration of infection is a well-described risk factor for the progression of liver disease in HCV [13], and we can presume a longer duration of the infection in older individuals who contracted the infection at a young age via sharing the drug injection equipment. Furthermore, the major acceleration of fibrosis progression was observed after the age of 50 years. The role of ageing in fibrosis progression could be related to a higher vulnerability to environmental factors, as well as to the higher prevalence of diabetes and obesity in older individuals [25]. In our cohort, diabetes was more prevalent in group F3–F4 (7/46 patients, 15.2 % vs. 2/154 patients, 1.3%). All these patients had type 2 diabetes, and diabetes was in line with obesity (*p* = 0.039). Most of the patients with diabetes in our group were obese (7/9), and 2/9 had overweight. In the group of 48 obese patients in the cohort, 9.6% had diabetes in contrast with non-obese patients, in whom diabetes was present only in 2.6% of individuals. 

In our cohort, harmful drinking also turned out to be a significant predictor of significant liver fibrosis; patients who self-reported harmful drinking had a 2.83× higher chance of suffering from significant fibrosis. Llamosas-Falcón et al. described that alcohol use has a dose-dependent relationship with incident cirrhosis in HCV patients and that the risk of disease progression rises already at low levels of alcohol consumption [24]. The overwhelming majority of HCV patients are unaware of their HCV status. Therefore, alcohol consumption is an unintentional risk factor for disease progression. Furthermore, some individuals who successfully stop illicit drug use can switch to harmful drinking, unaware of the diagnosis of hepatitis C. 

In our cohort of patients, 48 (24.0%) were obese, with a significantly higher proportion of obese patients in group F3–F4. The BMI of the patients who reported recent or ongoing drug use did not differ significantly from the BMI of patients who were abstaining on a long-term basis.

Obesity was the strongest factor predicting advanced liver fibrosis (OR 4.77) in our cohort. This finding is supported by recent data by Migdal et al. [26]; together with diabetes and liver steatosis, obesity significantly contributed to fibrosis progression in a cohort of 960 HCV treatment-naïve, unselected patients. In substance-abuse rehabilitation programmes, weight gain is common as a side effect of the medication for opioid-use disorder and may contribute to the patients’ stigma [27]. Baykara et al. reported an average weight gain of 4.6 kg in the first four months in patients in the buprenorphine/naloxone maintenance programme [28]. Participants of rehabilitation programmes also report a more structured lifestyle with regular food intake, but during the early stages of recovery, they report eating large amounts of food and cravings for sweets and junk food, which often serve as a replacement for drugs [29].

In Europe, two-thirds of the HCV disease burden is attributable to unsafe injection drug use [5]. The goal of HCV therapy in PWID is not only to prevent the progression of liver disease and its complications but also to prevent the onward transmission of the infection [12]. Owing to the high prevalence of HCV infection, PWID represent a population of paramount importance for enhanced screening and priority treatment [7]. A nationwide programme of the treatment of all patients with hepatitis C (TraP Hep C, Treatment as Prevention) was launched in January 2016 in Iceland. Universal access to direct-acting antiviral treatment was provided in order to treat the majority of HCV-positive individuals within two years from the start of the programme, with an emphasis on active PWID with a short history of HCV infection [30]. During the first three years, 95.3% of patients with a diagnosed infection were referred to therapy, and anti-HCV treatment was started in 96.5% of these patients. The SVR was achieved in 90.2% of patients. The programme led to a decrease of 82% in the prevalence of HCV RNA positivity in the active PWID in Iceland [31]. 

Even in patients with ongoing drug use, anti-HCV therapy has been demonstrated to be highly effective and safe [18,19,20]. In the Simplify study, participants reported ongoing drug use—the SVR12 rate was 94% (97/103 patients were cured). Only 6% and 1% of patients reported grade 3 and grade 4 adverse events, respectively, up to 28 days after the last dose of the study medication, a sofosbuvir and velpatasvir combination [19]. In the ANCHOR study, one hundred patients were treated with sofosbuvir and velpatasvir combination for 12 weeks; their treatment was co-localised with buprenorphine opioid substitution therapy in a drop-in centre in Washington, DC. Eighty-two patients achieved an SVR. The SVR achievement was not associated with baseline opioid-substitution therapy, on-treatment drug use, or poor drug adherence. The only factor associated with SVR achievement was the completion of the entire contents of two or more study drug bottles (each containing 28 pills) [18].

In our cohort, 183 (91.5%) patients in whom HCV RNA was checked 12 weeks after the end of therapy achieved SVR and confirmed an acceptable SVR rate in this difficult-to-treat patient group. The treatment was tolerated well, and none of the patients reported serious adverse events or required hospitalisation.

The risk of reinfection in individuals with ongoing drug use is still a matter of debate trying to justify the delay in anti-HCV treatment initiation caused by mandatory proven long-term abstinence or at least good compliance in opioid substitution therapy. However, recently published studies showed that these restrictions are unsubstantiated [11,18,19,32]. Real-world data support early treatment initiation, including therapy in people with ongoing substance use. The Trap Hep C programme results show reinfection in 50 patients (50/614, 8.1%), corresponding to a reinfection rate of 7.7 per 100 person-years. The risk factors of reinfection were younger age and recent drug use, defined as drug use within six months of the baseline visit to the treatment centre [33]. Although the authors consider the reinfection rate high, it is acceptable regarding high treatment efficacy leading to a sharp decrease in HCV prevalence in the PWID community. 

All patients motivated to enter therapy should start treatment betimes, supported in harm reduction measures to reduce the risk of reinfection [20,32]. The opioid substitution therapy leads to a more than 50% reduction of new HCV acquisitions. It is enhanced in people participating in further harm reduction strategies, such as needle and syringe exchange programmes [34]. Cooperation of patients with low threshold centres and peer programmes contributes to harm reduction awareness and decreases the risk of reinfection after a successful course of therapy [11]. 

The willingness of the patients to initiate anti-HCV treatment and their cooperation with harm reduction services is a crucial factor when considering treatment in PWID; postponing therapy until the time when long-term abstinence has been achieved can contribute to the disease progression and lead to a further spread of HCV infection in the PWID community. Nowadays, PWID represent the most important group of patients in implementing the WHO HCV elimination plan by 2030 [35], provided that all diagnosed patients are treated without restrictions concerning the duration of abstinence or the stage of liver fibrosis [36]. Early initiation of antiviral therapy is also dominantly cost-effective, less expensive, and more effective than delayed treatment initiation [37], preventing the progression of liver disease and the need for life-long medical care. 

Owing to the low prevalence of HCV infection in the Czech Republic, population screening for HCV infection has not been adopted [2]. However, enhanced screening in high-risk populations is well established and includes PWID who start harm reduction measures or addiction therapy, people who are admitted to psychiatric hospitals, and imprisoned individuals. Once tested positive, they are immediately linked to care; 90% of individuals who are diagnosed with HCV in prison start their antiviral treatment there [38].

According to current guidelines, assessment of liver disease severity by non-invasive tests is recommended before HCV treatment initiation to identify individuals with advanced liver disease who should remain in further close follow-up [12,17]. Patients with significant liver fibrosis achieving HCV cure should enter HCC surveillance programmes. However, ultrasound screening suffers from poor adherence. Recently published data showed that the adherence rate to biannual screening ultrasound in high-risk individuals was only 24% [39]. Twenty-eight patients with F3 and F4 in our cohort remained in the HCC surveillance programme. Their adherence was surprisingly high; only 32.1% of patients abandoned their follow-up at a median of 24 months. 

## 5. Conclusions

Our study showed a significant prevalence (23%) of patients with severe fibrosis or cirrhosis in the group of PWID initiating antiviral therapy of chronic hepatitis C. The factors predicting the presence of significant fibrosis were obesity, abstinence from illicit drugs for more than one year, harmful drinking, and older age. 

Our data stress the need for effective screening of HCV infection in the population of PWID and their early referral to antiviral therapy, with a particular emphasis on individuals with obesity and a history of harmful drinking. HCV treatment should represent an integral component of illicit drug use weaning programmes, regardless of patients’ age. Patients with a history of HCV infection should be aware of the need for early antiviral treatment and of the risk of liver disease progression if they suffer from obesity and harmful drinking. The healthcare workers should not only screen for HCV infection in the at-risk population but also provide support in the area of alcohol consumption and a healthy diet.

## Figures and Tables

**Table 1:** HCV treatment regimens and treatment efficacy.

	F0–F2 Group (N = 154)	F3–F4 Group (N = 46)	*p*
Direct-acting antivirals combination			
Glecaprevir + pibrentasvir	63 (41.0%)	4 (8.8%)	N.A.
Paritaprevir/ritonavir + ombitasvir + dasabuvir	38 (24.7%)	11 (23.9%)	
Sofosbuvir + ledipasvir	20 (13.0%)	5 (10.9%)	
Grazoprevir + elbasvir	9 (5.8%)	10 (21.7%)	
Sofosbuvir + velpatasvir	21 (13.6%)	12 (26.1%)	
Sofosbuvir + velpatasvir + voxilaprevir	1 (0.6%)	2 (4.3%)	
Other	2 (1.3%)	2 (4.3%)	
SVR achievement (SVR12)	141 (92%)	42 (91.4%)	N.S.
HCV relapse	1 (0.6%)	2 (4.3%)	
Lost to follow-up	13 (8.4%)	2 (4.3%)	
Known reinfection	1 (0.6%)	0 (0%)	

N.A. not applicable; N.S. not significant; SVR, sustained virological response; SVR12, SVR at week 12 after the end of therapy.

**Table 2:** Patients’ characteristics.

	Group F0–F2 (N = 154)	Group F3–F4 (N = 46)	*p* Value
Male sex	97 (63.0%)	37 (80.4%)	0.032
Age (years, median, range)	35 (25–62)	45 (32–60)	<0.0001
BMI (kg/m^2^, median, range)	25 (18–35)	30 (19–42)	<0.0001
Obesity (BMI > 30)	25 (16.2%)	23 (50.0%)	<0.0001
ALT (IU/L, median, range)	82 (17–807)	136 (15–431)	0.0013
GGT (IU/L, median, range)	55 (10–1220)	166 (33–1138)	<0.0001
Baseline HCV RNA (IU/L, median, range)	512,000 (35–20,100,000)	1,305,000 (5020–13,000,000)	0.028
HCV genotype			0.005
1a	55 (35.7%)	8 (17.4%)	
1b	51 (33.1%)	25 (54.3%)	
3	46 (29.9%)	10 (21.7%)	
Other or unknown	2 (1.3%)	3 (6.6%)	
LSM (kPa, median, range)	6.1 (2.6–9.7)	14.3 (10–75)	<0.0001
CAP (dB/m, median, range)	242.5 (100–377)	276.5 (172–400)	0.0013
Fibrosis stage (Metavir Score)			N.A.
F0	41 (26.6%)	0 (0%)	
F1	59 (38.3%)	0 (0%)	
F2	54 (35.1%)	0 (0%)	
F3	0 (0%)	16 (34.8%)	
F4	0 (0%)	30 (65.2%)	
History of diabetes	2 (1.3%)	7 (15.2%)	0.0006
History of cirrhosis decompensation	0 (0%)	8 (17.4%)	N.A.
HBV co-infection	1 (0.65%)	0 (0%)	N.S.
Illicit drug use status			0.0017
Recent or ongoing drug use	42 (27.3%)	3 (6.6%)	
Abstinence > 1 year	44 (28.6%)	10 (21.7%)	
Abstinence > 5 years	68 (44.1%)	33 (71.7%)	
Opioid substitution therapy			N.S.
Methadone	0 (0%)	1 (2.2%)	
Buprenorphine	6 (3.9%)	1 (2.2%)	
Harmful drinking history	26 (16.9%)	18 (39.1%)	0.0023
Alcohol addiction treatment history	11 (7.1%)	8 (17.4%)	0.044
Imprisonment (yes)	23 (14.9%)	5 (10.9%)	N.S.
Tattoo (yes)	88 (57.1%)	24 (52.2%)	N.S.
Smoking (yes)	118 (76.6%)	30 (65.2%)	N.S.
Psychiatric comorbidity (yes)	35 (22.7%)	15 (32.6%)	N.S.
Reference to therapy			N.S.
Infectious diseases specialist	40 (25.9%)	17 (36.9%)	
Gastroenterologist	31 (20.1%)	9 (19.6%)	
General practitioner/other specialist	13 (8.5%)	7 (15.3%)	
Addiction specialist, psychiatrist	32 (20.8%)	4 (8.7%)	
Self-reference	38 (24.7%)	9 (19.5%)	
Education			N.S.
Primary	51 (33.1%)	12 (26.1%)	
Secondary, qualified worker	70 (45.5%)	22 (47.8%)	
Secondary, leaving examination	23 (15.0%)	9 (19.6%)	
University	2 (1.3%)	0 (0%)	
Unknown	8 (5.1%)	3 (6.5%)	

BMI, body mass index; ALT, alanine-aminotransferase; GGT, gamma-glutamyltransferase; LSM, Liver Stiffness Measurement; CAP, Controlled Attenuation Parameter; N.A., Not Applicable; N.S. Not Significant.

## Data Availability

The datasets used and analysed in the study are available from the corresponding author by request.
